# Tumor Microenvironment Varies under Different TCM *ZHENG* Models and Correlates with Treatment Response to Herbal Medicine

**DOI:** 10.1155/2012/635702

**Published:** 2012-05-29

**Authors:** Zhen Chen, Lian-Yu Chen, Peng Wang, Hai-Yan Dai, Song Gao, Kun Wang

**Affiliations:** ^1^Department of Integrative Oncology, Fudan University Shanghai Cancer Center, Shanghai 200032, China; ^2^Department of Oncology, Shanghai Medical College, Fudan University, Shanghai 200032, China

## Abstract

In traditional Chinese medicine (TCM), diagnosis of pathology and choice of treatment prescriptions are based on a method of differentiation of signs and symptoms known as syndrome differentiation or *ZHENG*. The cornerstone of TCM, *ZHENG*, relies on the gathering of clinical information through inspection, auscultation and olfaction, inquiry, and palpation. However, the biomolecular basis of the *ZHENG* remains unclear. In this study, we established mouse xenograft pancreatic cancer models with *Shi-Re* (Dampness-Heat), *Pi-Xu* (Spleen-Deficiency), or *Xue-Yu* (Blood-Stasis) *ZHENG*, which are regarded as the three major *ZHENG*s in pancreatic cancer. We found that tumors of the different *ZHENG* models exhibited significantly altered cancer-associated fibroblast (CAF) proliferative activity and tumor-associated macrophage (TAM) infiltration, which led to altered levels of CAF- and TAM-derived secreted cytokines such as SDF-1 and CCL5. The *ZHENG* model type also significantly influenced tumor growth, and administration of herbal medicine to the *ZHENG* model modified the tumor microenvironment. Therefore, this study partially unveiled the molecular basis of TCM *ZHENG* in pancreatic cancer.

## 1. Introduction

Traditional Chinese medicine (TCM) has a history of over 3000 years. A holistic form of medicine, TCM, emphasizes bringing the patient's body, mind, and spirit into harmony. The theory and application of TCM are one of constant summarizing, inducing, and refining of the experiences accumulated in preventing and treating diseases in daily life and medical practice.

TCM rests squarely on *ZHENG* (syndrome) differentiation, a process of analyzing data collected through four combined diagnostic methods: inspection, auscultation and olfaction, inquiry, and palpation. All diagnostic and therapeutic methods in TCM are based on the differentiation of *ZHENG*. In modern times, TCM has become popular worldwide because of its reliable therapeutic efficacy [1]. However, diagnosis in TCM depends on the intuition and experience of the physician grounded in TCM theory, and this method seems to lack objectivity, accuracy, and reproducibility in the face of biomolecular science and Western-based medicine. Furthermore, the concept of *ZHENG* is often misinterpreted and unclear. For all these reasons, researchers from China and elsewhere have begun to investigate the *ZHENG* of TCM for a molecular foundation.

Tumors are now recognized as structures of multiple cell types, comparable to organs in complexity, which during tumorigenesis recruit the involvement of surrounding normal cells to construct and interact within a tumor microenvironment [2]. Continuous paracrine signaling with feedback within this microenvironment eventually leads to the end stages of cancer progression [3]. As cancer is no longer considered a discrete entity defined only by the traits of cancer cells within the tumor but may eventually involve the entire organism, TCM offers a holistic approach whose goal is regulating the integrity of all body functions as well as the interaction between the human and surrounding environment.

We have previously shown that the presence of *ZHENG* may influence tumor growth in pancreatic cancer. We also found that this effect might correlate with the CC chemokine (*β*-chemokine) family [4]. This finding suggests an involvement between *ZHENG* and the tumor microenvironment and deserves further research. Accordingly, in the present study we evaluated the tumor microenvironment under different *ZHENG* conditions, specifically noting changes in the proliferative activity of cancer-associated fibroblasts (CAF) and the infiltration of tumor-associated macrophages (TAM). We confirm here that characteristics of the tumor environment correlated with the *ZHENG* of TCM, and herbal treatments modified the tumor microenvironment.

## 2. Materials and Methods

### 2.1. Cell Lines and Mice

Samples of the pancreatic cancer cell line Panc02 were obtained from the Cancer Research and Development Center and grown in complete growth medium as recommended by the manufacturer. The cultured cells were maintained in a humidified 5% CO_2_ atmosphere at 37°C. All cells were regularly authenticated by observing cell morphology and tested for the absence of mycoplasma contamination using a MycoAlert Mycoplasma Detection kit (MycoAlert, Lonza, Rockland, ME, USA).

Male C57 mice, 4- to 6-week old, were obtained from the Shanghai Institute of Materia Medica at the Chinese Academy of Sciences (Shanghai, China) and housed in laminar flow cabinets under specific pathogen-free conditions with food and water supplied *ad libitum*. All experiments on mice were conducted in accordance with the guidelines of the National Institutes of Health (NIH) for the Care and Use of Laboratory Animals. The Committee for the Use of Live Animals in Teaching and Research, Fudan University, Shanghai approved the study protocol.

### 2.2. Drugs and Reagents

Individual packets of herb powders for each herb were produced by Jiangyin Tianjiang Pharmaceutical. The final decoction of each prescription was prepared at the Department of Pharmacy, Fudan University Shanghai Cancer Center, Shanghai, China, by dissolving the herb powder into distilled water to the required concentration. The daily dosage of herb decoctions for the mice was calculated according to the following human-mouse transfer formula: *Db* = *Da* × (*Rb*/*Ra*)×(*Wb*/*Wa*)  2/3, where *D*, *R*, and *W* represent dosage, shape coefficient, and body weight, respectively, and *a* and *b* represent human mouse, respectively.

 Honey supplied by Guan Sheng Yuan International Trade (Shanghai) was adjusted to a concentration of 20% in water. Wine (er guo tou) obtained from Hongxing (Beijing) was diluted to 55% in water. Pork fat was donated by Gu Jianzhong, Chinese Academy of Sciences (Shanghai). The following antibodies were used: anti-vimentin, anti-*α*-smooth muscle actin (SMA), anti-C-X-C chemokine receptor type 4 (CXCR4), and anti-C-C chemokine receptor type 5 (CCR5; all from Epitomics), and anticluster of differentiation 68 (CD68; Santa Cruz Biotechnology, Santa Cruz, CA).

### 2.3. Establishment of TCM *ZHENG* Model

We developed three types of TCM *ZHENG* models in respective mouse groups, namely, *Shi-Re* (Dampness-Heat), *Pi-Xu* (Spleen-Deficiency), and *Xue-Yu* (Blood-Stasis). The *Shi-Re* and *Pi-Xu ZHENG* models were established as we described previously [4]. Briefly, the *Shi-Re* condition was established by the wine and the pork fat combination (day 1 to day 7, 0.2 mL), and the food and honey-water were provided. *Pi-Xu* was developed by feeding the mice with a decoction of mirabilite and Chinese rhubarb, 0.2 mL for each mouse (day 1 to day 7). The *Xue-Yu ZHENG* was established by subcutaneous injection of 0.01% adrenaline (0.13 mg/kg) for each mouse (day 1 to day 7), as we described previously [5].

### 2.4. Subcutaneous Xenograft Tumor Model

Panc02 cells (2 × 10^6^ cells in 200 *μ*L) were injected subcutaneously into the right axilla of each C57 mouse. The length and width of tumors (in millimeters) were measured weekly with calipers. Tumor volume was calculated by the formula (*a* × *b* ^2^) × 0.5, where *a* and *b* were the long and short dimensions, respectively. Mice were euthanized under anesthesia when tumors reached 1.5 cm in diameter. The tumors were then resected and weighed. Each group had ≥10 mice.

### 2.5. Immunohistochemical Analysis

Specimens of tumor tissue were fixed in 10% formalin and embedded in paraffin wax. Unstained 3 *μ*m sections were then cut from paraffin blocks for immunohistochemical (IHC) analysis. The sections were stained with rabbit anti-vimentin (1 : 100), rabbit anti-*α*-SMA (1 : 100), rabbit anti-CD68 (1 : 200), rabbit anti-CXCR4 (1 : 200), and rabbit anti-CCR5 at 4°C overnight. The secondary antibody and avidin-biotin peroxidase complex method was used according to the standard protocols provided by the manufacturer (Vector Laboratories, CA, USA). An immunoglobulin-negative control was used to rule out nonspecific binding. Two independent assessors and one pathologist performed all procedures, all of whom were blinded to the model/treatment type for this series of specimens.

To quantitatively evaluate the CAF proliferative activity and TAM infiltration in each group, we calculated the ratio of the area positive for vimentin or CD68 staining to the total area in histological sections from ten fields under light microscopy (200x). The procedure for evaluation of CXCR4 and CCR5 expression followed that of our previous report [4].

### 2.6. Enzyme-Linked Immunosorbent (ELISA) Assay for Cytokine Release

The concentrations of SDF-1 and CCL5 in the tumor samples were determined using a sandwich ELISA kit (DuoSet; R&D Systems, Minneapolis, MN) according to the protocol of the manufacturer. Briefly, frozen tumor tissue was homogenized in lysis buffer and thereafter centrifuged at 12,000 rpm for 15 minutes at 4°C; 50 *μ*L of the supernatant was used for ELISA. Concentrations of immunoreactive SDF-1 and CCL5 were expressed as pg/mL.

### 2.7. Statistical Analyses

The data are expressed as the mean ± standard error (SE) of three or more independent experiments performed in triplicate. The statistical analyses were performed using analysis of variance (ANOVA) models and Student's *t*-tests. A *P* value <0.05 was accepted as statistically significant.

## 3. Results

### 3.1. *ZHENG* Distribution in Pancreatic Cancer Patients

**Figure 1:**
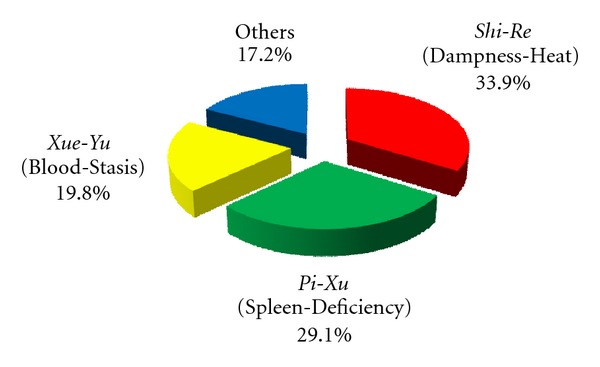
Percentages of pancreatic patients diagnosed with various TCM *ZHENG* conditions. Data on *ZHENG* distribution were extracted and analyzed from 34 clinical articles published between January 1, 1998 and December 31, 2008.

We firstly investigated the distribution of *ZHENG* conditions in populations of pancreatic cancer patients based on reports published from January 1, 1998 to December 31, 2008. Sixty-nine studies were identified by electronic and hand searches, among which 34 clinical articles were included for our study. Data on *ZHENG* distribution were extracted and analyzed. Twenty-seven *ZHENG*s were identified. The three *ZHENG*s in pancreatic cancer that were most reported were Dampness-Heat (in Chinese, *Shi-Re*; 33.9% of studies), Spleen-Deficiency (*Pi-Xu*; 29.10%), and Blood-Stasis (*Xue-Yu*; 19.8%; Figure 1).

### 3.2. Alteration of Tumor Microenvironment under Different *ZHENG* Conditions

The tumor microenvironment plays an important role in the development and progression of cancer [6, 7]. Pancreatic carcinomas are surrounded by desmoplastic stroma consisting of fibroblasts, immune cells, endothelial cells, and pericytes [8]. We hypothesized that the tumor microenvironment would be altered under different *ZHENG* conditions. To verify this hypothesis, we first established 3 subcutaneous tumor models of pancreatic cancer in mice that exemplified the *ZHENG* conditions *Shi-Re*, *Pi-Xu*, and *Xue-Yu*, respectively. We sought to investigate the differences in the tumor microenvironment among these *ZHENG* models.

**Figure 2:**
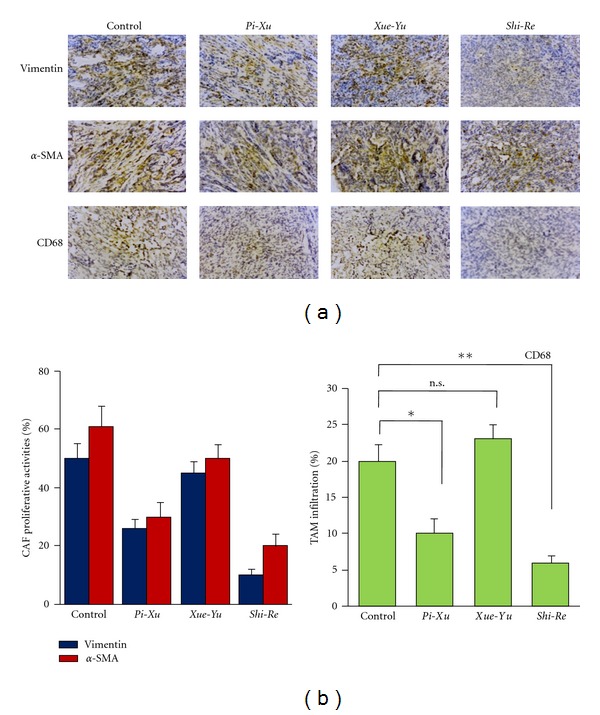
Alterations of tumor microenvironments under different *ZHENG* conditions. (a) Xenograft tumor models were established as described. On the next day, three types of *ZHENG*, namely, *Shi-Re*, *Pi-Xu*, and *Xue-Yu* were established. Tumors were obtained 4 wks after implantation. IHC staining for vimentin and *α*-SMA on sections of tumors was performed for evaluating CAF proliferative activities (top). IHC staining for CD68 was performed for evaluating TAM infiltration (low). Original magnification, 200x. (b) CAF proliferative activity (left) and TAM infiltration (right) were quantitatively evaluated by calculating the ratio of vimentin or CD68 antibody positive staining area to the total area in each field, and the mean value from ten fields under 200x microscopy was indicated. *P < 0.05; **P < 0.01; n.s.: not significant.

As it is recognized that in many tumors the stroma is characterized by an increase in fibroblast proliferation, we immunostained CAFs using the fibroblastic marker vimentin combined with the defined myofibroblast marker *α*-smooth muscle actin (*α*-SMA) to investigate the proliferative activity of CAF [9]. We found that the number of both vimentin- and *α*-SMA-positive cells was decreased in tumors from the *Shi-Re* and *Pi-Xu ZHENG* models of pancreatic cancer compared with the control tumor, while tumors from the *Xue-Yu* model exhibited no changes in CAF activity (Figures 2(a) and 2(b)). This observation suggested that CAF proliferative activity in tumors was altered differently on the basis of *ZHENG* conditions.

It is accepted that, in general, cancer- and host-cell-derived signals program TAMs to acquire an M2-like polarized and otherwise tumor-supportive phenotype [10]. In many cases increased numbers of TAMs are associated with a poorer prognosis [11, 12]. Therefore, we evaluated tumors in the different *ZHENG* models for TAM infiltration by staining for CD68 (also known as macrosialin in mice), a glycoprotein expressed on macrophages. We found that, compared with the control group, the number of macrophages was dramatically less in the *Shi-Re* group, followed by lesser degrees of decrease in the *Pi-Xu* groups (Figures 2(a) and 2(b)). This observation suggests relatedness between the inflammation characteristics of tumor microenvironments and the specific *ZHENG* conditions tested. Altogether, our study demonstrated a correlation between *ZHENG* conditions and the microenvironment of tumors in pancreatic cancer.

### 3.3. Correlation between Tumor Microenvironment and Growth under Different *ZHENG* Conditions

**Figure 3:**
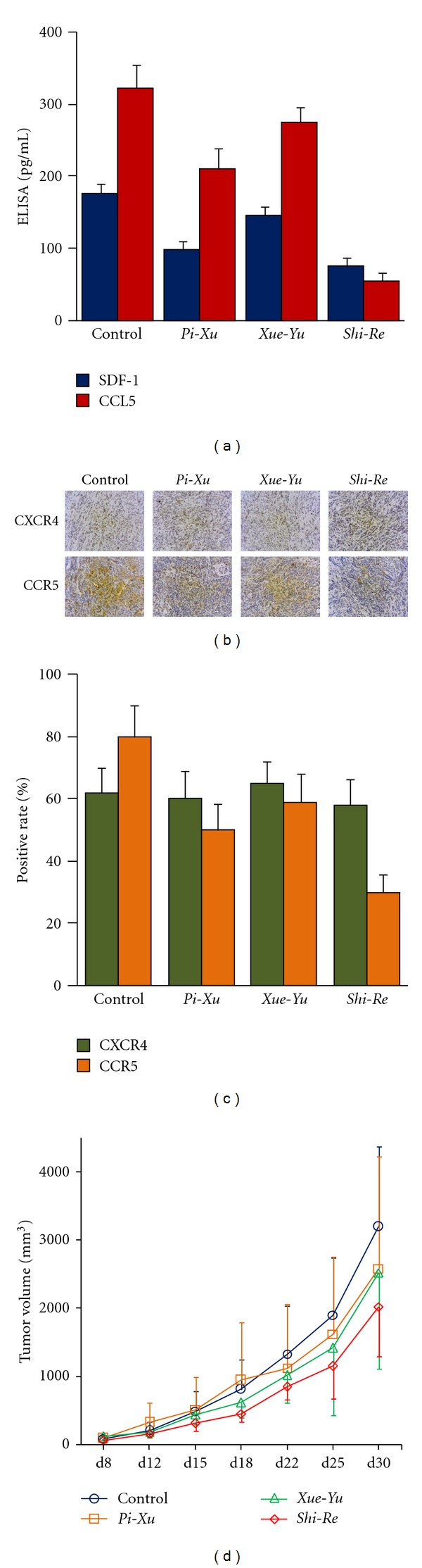
Correlation between microenvironment and tumor growth under different *ZHENG* conditions. (a) The levels of CAF- and TAM-derived secreted cytokines, SDF-1 and CCL5, in tumor from the *Shi-Re*, *Pi-Xu*, and *Xue-Yu ZHENG* models were evaluated with ELISA assay. Data are expressed as the mean ± SE. (b and c) IHC staining for CXCR4 and CCR5 on sections of tumors from *ZHENG* models was performed and representative photos are shown in (b). The positive rates of CXCR4 and CCR5 protein in tumors with indicated *ZHENG* are shown in (c). (d) Effect of *ZHENG* on tumor growth in a subcutaneously transplanted tumor model. Xenograft tumor model combined with *ZHENG* model was established as described in Figure 2(a). Mean volumes of tumors from each group were measured. Mean ± standard deviation was determined for 10 mice in each group.

CAFs stimulate tumor cell proliferation and invasion through various growth factors, hormones, and cytokines [13]. SDF-1 is a CAF-derived chemokine that has been shown to directly boost the proliferation and invasion of pancreatic cancer cells [14]. Thus, we evaluated the levels of secreted SDF-1 in tumors under different *ZHENG* conditions, and the expression of CXCR4, the SDF-1 cognate receptor, in tumor cells. The results of ELISA assays showed decreased levels of SDF-1 released in tumors in the *Shi-Re* and *Pi-Xu* groups compared to the control mice. This was not observed in the *Xue-Yu* tumors (Figure 3(a)). This result was consistent with the observation that the *Shi-Re* and *Pi-Xu* tumors exhibited decreased CAF proliferative activity. However, there was no difference in CXCR4 expression among the *ZHENG* models and control tumor cells (Figures 3(b) and 3(c)).

Similarly, we wanted to verify whether the decreased TAM infiltration we observed above led to a reduction in the levels of the TAM-derived cytokine CCL5. We found that secreted CCL5 decreased dramatically in tumors under *Shi-Re* and *Pi-Xu ZHENG*s. This was also observed in the *Xue-Yu* tumors, although the difference was not significant (*P* = 0.083) (Figure 3(a)). We also found that tumor cells from the *Shi-Re*, *Pi-Xu,* and *Xue-Yu* models exhibited decreased CCR5 expression, especially for *Shi-Re* (Figures 3(b) and 3(c)).

After we confirmed that CAF-related SDF-1/CXCR4 and TAM-related CCL5/CCR5 expressions were changed under different *ZHENG* conditions, we next investigated an association between the altered tumor microenvironments and tumor growth. We found that altered tumor microenvironments were correlated with *in vivo* changes in tumor growth (Figure 3(d)). Taken together, these results suggest that tumors under different *ZHENG* conditions exhibited different tumor microenvironments, which may finally effect tumor growth.

### 3.4. Tumor Response under Different *ZHENG* Conditions to Herbal Medicine Treatments

**Figure 4:**
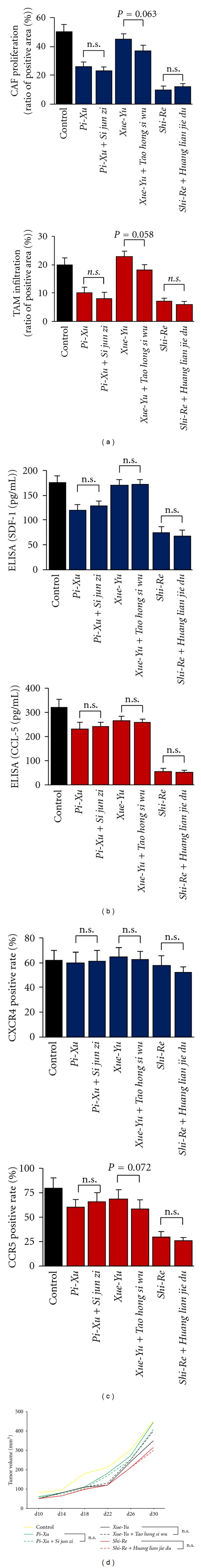
Response of tumors under different *ZHENG* conditions to herbal medicine treatments. (a) IHC staining for vimentin, *a*-SMA, and CD68 in tumors from the indicated group was performed for evaluating CAF proliferative activities and TAM infiltration, respectively. CAF proliferative activity and TAM infiltration were quantitatively evaluated as described in Figure 2(b). (b) The levels of SDF-1 and CCL5 in indicated tumors were evaluated with ELISA assay. Data are expressed as the mean ± SE. (c) IHC staining for CXCR4 and CCR5 on sections of indicated tumors. The positive rates of CXCR4 and CCR5 protein in tumors with indicated *ZHENG* was calculated. (d) Effect of herbal medicine on subcutaneously transplanted tumor with different *ZHENG*. The growth curves for each are shown. n.s.: not significant.

TCM usually means a comprehensive assessment of pathogenesis, location, and disease pathology, and the diagnosed *ZHENG* helps guide the application of Chinese herbal remedies. So, we used *Huang lian jie du* decoction (a traditional prescription used of treating *Shi-Re ZHENG*), *Si jun zi *decoction (a traditional prescription used of treating *Pi-Xu ZHENG*), and *Tao hong si wu* decoction (a traditional prescription used of treating *Xue-Yu ZHENG*) for *Shi-Re*, *Pi-Xu,* and *Xue-Yu* tumors, respectively. The herbal prescriptions used are shown in Table 1. We found that the herbal medicines had no or little effect on CAF proliferation or TAM infiltration (Figure 4(a)). We also evaluated the effects of the herbal medicines on SDF-1 secretion and CCL5 levels and found that the levels of which did not change after herbal medicine treatment (Figure 4(b)). Similarly, none of the herbal medicines had significant effects on CXCR4 or CCR5 expression in tumor cells (Figure 4(c)). These observations indicated that the herbal medicines had little effect on either the tumor or microenvironment. Finally we evaluated the effects of the herbal medicines on tumor growth and could not find any difference when the tumors were treated with different types of herbal medicine (Figure 4(d)). Therefore, our results suggest that a prescription based solely on *ZHENG* does not always result in a satisfactory response.

### 3.5. Herbal-Medicine-Induced Alteration of Tumor Microenvironment Is Correlated with Treatment Response

**Figure 5:**
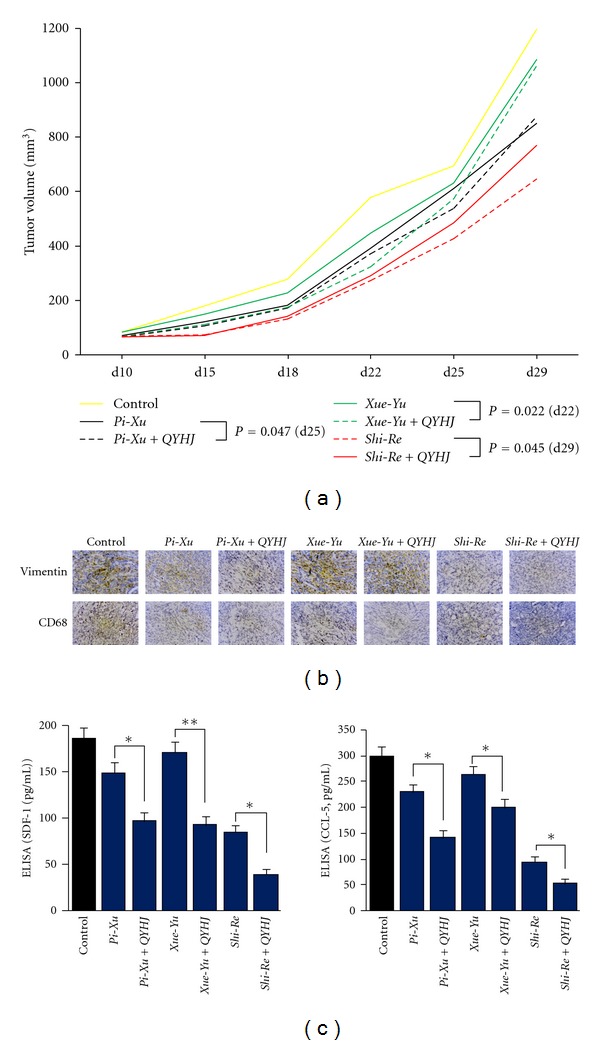
Treatment response to herbal medicine involved with modification of tumor microenvironment. (a) The antitumor effect of Qingyihuaji formula (QYHJ) on tumor with different *ZHENG*. (b) The effect of QYHJ on CAF proliferative activities and TAM infiltration were evaluated as described in Figure 2. (c) The effect of QYHJ on secreted SDF-1 and CCL5 levels were evaluated as described in Figure 3(a). *P < 0.05; **P < 0.01.

Although tumors under different *ZHENG* conditions demonstrated differences in tumor microenvironment which may finally be reflected in tumor growth, the *ZHENG* conditions themselves seemed not to promote tumor growth. Thus, herbal medicines prescribed on the basis of the *ZHENG* condition alone did not affect tumor growth. To further understand the relatedness of tumor microenvironment, *ZHENG*, and the response to herbal treatment, we employed the *Qingyihuaji* formula (QYHJ), a prescription based on TCM theory whereby pancreatic cancer is considered to be of *Shi-Re* origin. QYHJ has been used to treat pancreatic cancer for many years [15, 16]. We first established mouse tumor models with the accompanying *ZHENG* conditions *Shi-Re*, *Pi-Xu*, and *Xue-Yu*. The mice were then treated with QYHJ and the tumor microenvironment and tumor growth were evaluated. We found that tumors of the different *ZHENG* condition models exhibited altered tumor microenvironments (Figures 5(b) and 5(c)), which is consistent with our previous observations (Figure 2). The QYHJ treatments altered the tumor microenvironments in the *Shi-Re*, *Pi-Xu*, *Xue-Yu* models dramatically, as demonstrated by decreased CAF proliferation and TAM infiltration (Figures 5(b) and 5(c)). Then when we correlated tumor microenvironment alteration with tumor growth, we surprisingly found that QYHJ treatment led to growth inhibition of tumors under different *ZHENG* conditions, although the inhibition rate varied among the different *ZHENG *(Figure 5(a)). This suggests that disease identification is sometime a requisite for the treatment of cancer with TCM. Taken together, these results indicate that a combination of disease diagnosis and *ZHENG* identification is essential for clinical TCM practice in cancer treatment. They also showed a relatedness between the tumor environment and *ZHENG*, and treatment response to herbal medicine involved the modification of the tumor microenvironment.

## 4. Discussion

In this study, we established three different *ZHENG* mouse models according to TCM theory. We identified alterations in the tumor microenvironment under different *ZHENG* conditions. These tumor microenvironment modifications mediated a correlation between the *ZHENG* condition and response to herbal treatment. Therefore our study revealed a molecular basis for *ZHENG* in pancreatic cancer.

In TCM clinical practice, *ZHENG* helps guide the remedy prescription and therefore has an important position in the TCM system, that is, *ZHENG* is the key to recognizing diseases and the foundation to treat them. However, because of the complexity of the concepts (e.g., a single *ZHENG* involves multiple anatomical systems) and lack of nonprofessional descriptions, research of *ZHENG* is difficult to advance. The molecular basis underlying *ZHENG* in TCM remains unclear.

It has been confirmed that tumor cells do not act in isolation, but rather subsist in a rich microenvironment provided by resident fibroblasts, inflammatory cells, endothelial cells, pericytes, leukocytes, and extracellular matrix [17]. It is increasingly appreciated that, as the cancer progresses, the surrounding microenvironment is activated in support, coevolving through continuous paracrine communication and supporting carcinogenesis [3]. Pancreatic ductal adenocarcinoma is characterized by an extensive stromal response called desmoplasia. Within the tumor stroma, CAFs are the primary cell type; the importance of the role of CAFs in tumor progression is now well accepted. CAFs produce large amounts of secreted factors, including CXC, CC chemokines, and other inflammatory mediators that promote the proliferation, invasion, and metastasis of cancer cells [18]. It is also accepted that large numbers of tumor-associated leukocytes infiltrate solid tumors, and TAMs represent a major and important component of these leucocytes, which are driven toward functions that support cancer progression and poorer prognosis [12]. Therefore the stromal elements of tumors hold prognostic, as well as response-predictive, information. Abundant targeting opportunities within the tumor microenvironment are continually identified [3].

As TCM sustains systematic theories and is a holistic approach to health, and our previous study has indicated a correlation between *ZHENG* and levels of cytokines related to CAF and TAM [4], we hypothesized a correlation between the tumor microenvironment and the *ZHENG* syndromes of TCM. Thus we evaluated the tumor microenvironment by immunostaining for CAF and TAM and surprisingly found differences in microenvironment alterations under different *ZHENG* conditions. Furthermore, the alterations in CAF proliferative activity and TAM infiltration led to changed levels of CAF- and TAM-derived secreted cytokines which finally affected tumor growth.

Based on TCM theory and clinical experience, patients with pancreatic cancer usually exhibit *Shi-Re*, *Pi-Xu*, or *Xue-Yu*, and respective herbal decoctions for removing heat and dampness (*Huang lian jie du*), reinforcing Qi and strengthening the spleen (*Si jun zi)*, and promoting blood circulation and removing blood stasis (*Tao hong si wu*) are always prescribed. However the efficacy of these remedies is not always satisfactory. In the current study, the application of decoctions of these herbal medicines had little effect on tumor growth or the tumor microenvironment. This observation seems to contradict the TCM theories of treating the same disease with different methods and treating different diseases with the same methods. There are many reasons that may account for the lack of response to the TCM treatments in the present study. One is that each prescription has its priority and focus, although they are within the same category for the same *ZHENG*. Another reason is that apart from the traditional relationship between disease and *ZHENG*, there may also exist analogous *ZHENG* in the same disease, which means that different patients who suffer from the same disease manifest the common basic *ZHENG* in spite of slight differences in their accompanying symptoms. Therefore, we can use a basic prescription with slight modifications to treat accompanying symptoms. In fact recent research emphasized the principle of analogous *ZHENG* existing in the same disease in TCM clinical practice [19], especially in cancer treatment. These two reasons may partially explain why the prescriptions based on *ZHENG* used in the present study had little effect on tumor growth.

The integration of disease diagnosis and identification of *ZHENG* have been widely used in cancer treatment [20]. Based on our previous studies, pancreatic cancer is characterized by dampness, heat, and toxicity and should be treated by removing heat and dampness, detoxification and resolving a mass [21]. According to this recognition, we recommend the QYHJ formula in the treatment of pancreatic cancer. The results of our clinical studies suggest that treatment with QYHJ resulted in prolonged survival time for patients with pancreatic cancer [15, 21]. Animal studies showed that QYHJ could inhibit the growth of subcutaneously transplanted pancreatic tumors in nude mice [16]. Just as we can see from this study, QYHJ had an effect on tumor growth and the tumor microenvironment, although the effect varied depending on the *ZHENG* type. Therefore, our study suggests an intrinsic disease-specific *ZHENG*, which should be considered during TCM practice. The study also indicated that the tumor microenvironment influences the tumor response to herbal medicine treatment.

In conclusion, our study showed alterations in the tumor microenvironment under different *ZHENG* conditions. We also confirmed a relatedness between the tumor environment and *ZHENG*, and herbal medicine treatments modified the tumor microenvironment. This study partially unveiled the molecular basis of TCM *ZHENG* in pancreatic cancer.

## Figures and Tables

**Table 1:** Herbal prescriptions used in this study.

Prescription	Contents (g)
*Huang lian jie du* decoction	Coptis root (9), baical skullcap root (6), amur corktree bark (6), cape jasmine fruit (9)

*Si jun zi* decoction	Ginseng (9), largehead atractylodes rhizome (9), poria (9), liquorice root (6)

*Tao hong si wu *decoction	Peach seed (9), safflower (6), Chinese angelica root (9), rehmannia root (12), rhizome of szechwan lovage (6), radix paeoniae rubra (9)

*Qingyihuaji *formula	Herba scutellariae barbatae (15), hedyotidis herba (15), amorphophallus konjac (15), coix seed (15), fiveleaf gynostemma herb (15), java amomum fruit (10)
